# Applying a dyadic outcomes approach to supporting older carers and care‐recipients: A qualitative study of social care professionals in England

**DOI:** 10.1111/hsc.13914

**Published:** 2022-07-20

**Authors:** Stacey Rand, Wenjing Zhang, Grace Collins, Barbora Silarova, Alisoun Milne

**Affiliations:** ^1^ Personal Social Services Research Unit (PSSRU) University of Kent Canterbury UK; ^2^ Centre for Health Services Studies (CHSS) University of Kent Canterbury UK; ^3^ School of Social Policy Sociology and Social Research (SSPSSR), University of Kent Canterbury UK

**Keywords:** assessment, carers, long‐term care, outcomes, quality of life, social care

## Abstract

There are an estimated 2 million older carers, aged 65 or over, in the UK. Older carers are more likely to care for a co‐resident spouse/partner, provide high‐intensity support and have their own health problems. The literature suggests that a ‘dyadic outcomes approach’ to social care (i.e. services and support that seek to understand and improve the quality of life of the older carer and the person they support, individually and together) may be especially beneficial for older carers. Such an approach may be applied in needs assessment and review, service evaluation, planning and delivery, or commissioning. However, there is a paucity of evidence of its effectiveness and feasibility in practice. In this qualitative study, we explored views of social care professionals in England on supporting older carers, as well as the feasibility, potential benefits and challenges of applying a dyadic outcomes approach into policy and practice. Overall, 25 professionals were interviewed between January and July 2021, including social workers, team leads, managers, commissioners and other representatives from local authorities, care providers and carer organisations. Findings indicate that there is limited focus on the specific needs of older carers in practice. Participants recognised the potential benefits of a dyadic approach, including the development of a holistic view that enables an effective response to supporting quality of life, for both carer and care‐recipient, and building trust when working to support the caring dyad. Barriers to applying a dyadic approach included data protection and sharing, both within and between organisations; required workforce skills, experience and knowledge; and insufficient and competition‐oriented adult social care funding that discourages collaborations between agencies. Despite the potential of the approach to improve the effectiveness of support for older caring dyads, these challenges need to be recognised and addressed if it is to be implemented.

**What is known about this topic?**

- Older carers, aged 65 or over, are the fastest growing group of carers in England
- Older carers are more likely to provide high intensity care and have their own health needs, yet are relatively unrecognised in policy and practice
- A dyadic outcomes approach has been proposed to better support older carers

**What this paper adds?**

- Social care professionals have mixed views on whether older carers have distinct support needs
- The perceived benefits of applying a dyadic outcomes approach included a more holistic understanding of people's support needs and building trust
- Challenges included sector‐wide issues (e.g. workforce, funding models), which need to be understood and addressed to effectively implement a dyadic outcomes approach in practice

## 1 INTRODUCTION

In the UK, the fastest growing group of unpaid carers, who provide help and support to a relative or close friend with care needs, are older carers aged 65 years or over. There are an estimated 2 million older carers in the UK, which doubled to 4 million during the early phase of the Covid‐19 pandemic (Age UK, 2021). Older carers are more likely to care for a spouse/partner, co‐reside with that person, engage in mutual caring (also known as co‐caring) and provide high‐intensity support. In addition, they often balance the demands of care‐giving with their own age‐related health problems and later life transitions, for example retirement (Henwood et al., 2019; McGarry & Arthur, 2001). Despite this, older carers remain relatively unrecognised in policy, research and practice (Larkin et al., 2019).

Across all age groups, carers, especially those who care intensively, are at increased risk of adverse health and quality of life (QoL) outcomes (Public Health England, 2021). This includes effects on physical and psychological health, the ability to sustain social relationships, engage in paid employment, voluntary work or leisure activities, and access healthcare or other public services (Farina et al., 2017; Stenberg et al., 2009; Stöckel & Bom, 2021; Totsika et al., 2017; Yoong & Koritsas, 2012). Internationally, there is increasing emphasis on supporting carers to maintain their health and wellbeing, as well as investment into services and support, for example breaks from caring to alleviate the strain of caring and to enable carers to engage in activities that support health, like attending medical appointments, or well‐being, like leisure or social activities; financial benefits to offset the financial impact of caring through loss of employment and/or increased living costs; and leave entitlements for working carers to support them to stay in employment around caring responsibilities (Brimblecombe et al., 2018; Spiers et al., 2021). Although there are different emphasises and approaches by country, it is widely accepted that the aim of long‐term care (known as ‘social care’ in the UK), which includes home care and specialist support services for carers, is to support and improve people's QoL, including carers (Netten et al., 2012; Rand et al., 2015).

The caring relationship between carer and care‐recipient (the ‘caring dyad’) is typically embedded in a pre‐existing long‐term familial or close personal relationship. However, long‐term care systems tend to view, assess and address the needs and outcomes (i.e. impact on care‐related QoL) of individuals, rather than also recognising the caring dyad (as a unit). Adults with care and support needs are often viewed separately from their carers. This overlooks the importance of caring relationships and the wider familial and social context. This individualised focus is commonly found in approaches to assessments of need and in care planning, review and service delivery; it is also reflected in local, regional and national policy and funding related decisions, for example commissioning services, in the UK and other European countries (Courtin et al., 2014). An individualised approach overlooks the centrality of the relationship in caring and the interdependence of needs and QoL between individuals in such relationships (Rand, 2020).

The existing individualised approach reflects how publicly funded long‐term care systems have developed over time. Recognition of carers' rights to needs assessments and support, as well as to be regarded on an equal footing to care‐recipients, has emerged later (if at all) than those accorded to care‐recipients (Courtin et al., 2014). In England, the Care Act (2014) introduced a statutory duty on local authorities (LAs) to assess the needs of carers and meet any eligible support needs, whereas the duty to do this for adults with care needs is long‐established (Rand & Malley, 2014). The Care and Support (Eligibility Criteria) Regulations 2015 formally define carers' ‘eligibility for support’ in terms of needs related to their QoL outcomes, for example ability to maintain nutrition, sustain personal relationships and engage in leisure activities. The importance of supporting carers to sustain health and wellbeing, as envisaged by the Care Act, has been affirmed in the 2021 adult social care white paper, ‘People at the Heart of Care’ (Department of Health and Social Care, 2021; Marczak et al., 2021).

Carers occupy an ambiguous status in relationship to legislation, policy and practice. Carers have been variously conceptualised as providers of care (*co‐workers* alongside formal services), recipients of care (*co‐clients*, who are eligible for support in their own right) and as free resources (carers willingly providing support to their relative on an unpaid basis) (Marczak et al., 2021; Rand & Malley, 2014; Twigg & Atkins, 1994). The Care Act explicitly seeks to reinforce carers' status as *co‐clients*, entitled to support and to be treated on an equal footing to care‐recipients. It proposes the adoption of a ‘whole family approach’ to assessing and meeting needs by developing a view of the person with support needs in the context of their household/family and coordinating support based on this holistic perspective (Department of Health and Social Care et al., 2014). In this study, we focus on a *dyadic outcomes approach*, which relates to ‘the whole family approach’, as outlined above, with a particular focus on the outcomes of care (i.e. improving or maintaining QoL) for *both care‐recipients and carers*, individually *and* together (Rand, 2020). The dyadic outcomes approach is especially relevant to needs assessment and care planning, but it may also be applied to service delivery, planning and resource allocation.

This approach has significant appeal because the needs of the (usually older) care‐recipient and older carer often intersect. As most older carers are embedded in a lifelong relationship and tend to share a home and daily life, care‐related QoL needs/outcomes tend to be intertwined, with the QoL of one member influenced by, and influencing, the QoL of the other (Rand et al., 2017). A shift to viewing the carer and care‐recipient as a ‘unit’ may have particular resonance for older caring dyads (Lloyd, 2019; O'Rourke et al., 2021). This was the starting point for the research reported in this paper, which involved interviews with social care professionals in England to explore their views on: (1) addressing needs and supporting the QoL of older carers; and (2) the acceptability and potential benefits, challenges and barriers of applying a dyadic QoL outcomes approach in practice.

## 2 METHODS

This qualitative study was one of three strands in a study of dyadic QoL outcomes approaches to support older care‐giving dyads. The findings of the other two strands, a scoping literature review and interviews with carers and care‐recipients, are reported elsewhere (Zhang et al., 2022). The research questions were: (1) what are social care professionals' views on the support needs and outcomes of older carers, in general and (2) what are professionals' views of the acceptability and potential benefits, challenges and barriers to applying a dyadic QoL outcomes approach in practice.

### 2.1 Study participants

The study aimed to recruit a minimum of 20 social care professionals, to include a mix of social workers and support workers involved in assessments and/or care planning, team leaders, strategic‐level or service delivery managers, commissioners and policy or funding professionals. The study was conducted with support from LAs (*n* = 3), care providers (*n* = 4) and carers' organisations (*n* = 7) in London, the South East and central England. In collaboration with these organisations, a total of 33 participants were identified and invited to participate in the study. Of these, eight declined to participate due to lack of time and/or competing priorities (*n* = 4 commissioners, *n* = 3 social workers, *n* = 1 services manager). In four cases, individuals nominated another staff member to complete the interview in their place because their professional experience and expertise was a better fit for the study. In all cases, it was stressed that participation was voluntary. Organisations were not informed which of their staff (if any) participated.

A total of 25 social care professionals were interviewed, either one‐to‐one (*n* = 19) or with two staff members from one organisation together (*n* = 3). The range of roles included senior management (CEO, senior services manager or director, *n* = 10), services manager or team leads (*n* = 7), LA adult social care staff, including social workers (*n* = 5) or related roles (apprentice social worker or older carers support worker [*n* = 2]) and commissioners (*n* = 1). All of the care providers and LAs offered dual support to care‐recipients *and* carers.

The professionals' roles involved the use of standardised outcome measures and/or consideration of care‐related QoL outcomes in practice. These were routinely part of needs assessment and reviews (*n* = 9); evaluation and monitoring of funding or commissioning of services, and/or applying for new grants or contracts (*n* = 10); and routine in‐house monitoring of services, either for service planning and delivery (*n* = 10) or providing feedback to service users as part of local accountability (*n* = 2).

### 2.2 Qualitative interviews

Semi‐structured interviews were conducted online using MS Teams between January and July 2021. The interviews lasted between 22 and 48 min. They were conducted by one researcher (the first author). Written or verbal consent (with written record) was obtained from all participants.

The interview topic guide covered: (1) an overview of the participant's professional background and current role; (2) their organisation's current key priorities and concerns with regard to supporting older carers; (3) the use of outcomes in practice (e.g. for service design and planning, commissioning and funding, oversight and management, needs assessment, review and care planning), including benefits and challenges; (4) the potential benefits, challenges and barriers to applying a dyadic outcomes approach in practice. The definition of a ‘dyadic outcomes approach’ was developed from the participant's response to (3). They were asked whether outcomes were collected and applied to support care‐recipients and carers, together, rather than individually. In some cases, this approach was already applied, so the respondent could reflect based on practice. If not, then the participant was asked to reflect on what this approach could potentially offer.

### 2.3 Data analysis

The analysis was conducted by four experienced qualitative researchers (Authors 1–4) using the framework analysis method (Gale et al., 2013; Ritchie & Spencer, 1994). Each interview was audio‐recorded and transcribed verbatim by a professional transcriber. Names of people, organisations and places were replaced with pseudonymised codes. An initial working analytical framework was developed from the topic guide and coding of the first three interviews. This initial framework was reviewed by the research team. One researcher (first author) initially coded all of the interviews in NVivo. Further codes and sub‐codes were added, inductively. The second author independently analysed 10 interviews, using the same process. The coding of the 12 remaining interviews was reviewed by the third and fourth authors. Any discrepancies or queries were discussed, until consensus was reached. NVivo was used to generate framework matrices to facilitate the process of charting. Interpretation of the interview data was conducted throughout the analysis process.

### 2.4 Ethics statement

Ethical approval for the study was given by the North West Liverpool Central Research Ethics Committee (Reference: 20/NW/0473/281639), with approval also from the Association of Directors of Adult Social Services (ADASS) and local research governance approvals from participating LAs.

## 3 FINDINGS

The findings presented in this paper focus on themes related to professionals' views on (1) supporting older carers and (2) applying dyadic outcome approaches.

### 3.1 Supporting older carers

Respondents were asked for their professional views about current priorities and concerns related to supporting older carers. Some interviewees expressed the view that the health and QoL‐related needs of carers are similar across age groups, so their professional priorities and concerns were the same for all carers, regardless of age.

There are universal similarities amongst carers of all age groups in the things you do, having to juggle priorities, and feelings of stress and anxiety, having to advocate for someone else.

(PS16 carer organisation)

However, some expressed the view that age‐related factors influenced the experience and nature of caring, based on their professional experience. For example, the increased incidence of mutual/co‐ caring:

… especially seventy‐five and older, we start to pick up, um, people that you would call mutual carers who are in caring roles where they've got comorbidities of their own.

(PS1 care provider)

Overall, issues related to age and ageing were not seen as primary concerns, although one interviewee reflected on how the concepts of ‘older‐hood’ and an ‘older adults sector’ may create cohesion and a shared language across partners, to stimulate policy development, service planning and delivery to meet the needs of older people (PS4, carers’ organisation). Some respondents considered the lack of focus on older carers to be related to the absence of data on the age profile of carers who use a service or access support.

I haven't got a sense of how many over sixty‐five carers we've got in the service. It's not the sort of data we regularly collect…

(PS20 social worker)

The lack of focus on older carers may also be due to organisational structure or process. Most respondents (except one, whose role is specifically to support older carers) indicated that their professional focus is on identifying and addressing needs, through the processes of an adult social care system that, in some areas (e.g. assessment teams, carers services), operate without reference to age.

…people don't tend to talk about the age of the carers so much. Obviously, there's common issues that come up—issues that come up are things like …—are they eligible for a payment, and can they use it to spend it on the thing that they want to spend it on? Those sorts of debates.

(PS20 social worker)

In particular, the interviewees from carers' organisations reflected on how service provision (aside from specialist support for young or young adult carers, aged up to 25 years) is typically universally available to all carers, regardless of age: *“the offer is open to all carers”* (PS14, carers’ organisation) and *“older carers can access any of our services”* (PS15, carers’ organisation). This reflects the current model of commissioning carers' organisations to provide generic ‘support for carers’, without further specialisation, even if some respondents noted a trend towards recognising the needs of carers with specific experiences and offering tailored support, for example support for end‐of‐life or recently‐bereaved carers.

At the same time, however, there was some recognition that universal carers' services may be failing to meet the specific needs of older carers.

For some older carers, there's a particular kind of view on what support they would want that maybe doesn't match particularly how services, like ours, are funded now… [funders] want short term interventions… whereas a lot of older carers want to come to a group and continue coming to that group on a monthly basis forevermore.

(PS7 carers’ organisation)

There was also recognition that age could intersect with other inequalities to marginalise or exclude an older carer from accessing a service:

I think for older carers you've kind of got multiple barriers going on. So for LGBT carers or BAME [Black Asian Minority Ethnic] carers, because there's concern or mistrust about what they might encounter in say a carer support group, in terms of prejudice or exclusion by other members of the group—even though obviously we aim to keep them as safe spaces.

(PS7 carers’ organisation)

### 3.2 Applying a dyadic outcomes approach

#### 3.2.1 Benefits

All respondents identified potential benefits of applying a dyadic outcomes approach. Due to the range of professionals interviewed in the study, these benefits related to different levels or perspectives within the long‐term care system, including needs assessment, care planning, service planning, delivery or monitoring, and commissioning or funding. The majority of respondents (*n* = 16) identified that such an approach, if applied flexibly and responsively, would allow a more holistic understanding of people's support needs (regardless of whether a ‘carer’ or ‘care‐recipient’) to inform care planning, service delivery and coordination, especially in the way(s) in which needs/outcomes are mutually interdependent within familial or close relationships.

I'm very supportive of that approach actually because what we find is you really do need that whole family approach to build up a picture of what's actually happening.

(PS14 carers’ organisation)

I feel strongly that a holistic approach to assessment will be useful, rather than separating and labelling them. Capture them in one I feel.

(PS21 social worker)

I think one of the opportunities of taking a wider view of things means that we can have much more coordinated approach around supporting people… having that joined up approach with an outcome focus, is only beneficial in the long run.

(PS24 commissioner)

Indeed, a number of respondents questioned the value of ‘labelling’ people, as ‘carers’ or ‘adults with support needs’. They challenged the bureaucratically generated and administrative nature of this terminology, which, they regarded as a constraint to thinking and the ability to creatively address people's needs in a flexible, contextual and personalised way.

In an ideal world… those services would be genuine and would be geared up for that cared for, and that carer. It'd be their package of care, not the cared for, or the carer, it would be their package of care.

(PS11 care provider)

A dyadic outcomes approach may also emphasise and focus professionals' awareness on how social care support interventions for one person in the caring dyad, whether carer or care‐recipient, may have an effect on the other person in the dyad.

Whatever support you give to a carer, it is going to have some kind of impact on the person that they're caring for. You would hope that would be a positive impact. It often will be.

(PS4 carers’ organisation)

Some professionals, especially those who worked for organisations that serve both carers and care‐recipients (i.e. LAs, care providers), expressed their views based on the practice of applying a dyadic outcomes approach in needs assessment, care planning and the provision of support. Where professionals were working with both care‐recipient and key family members (whether formally defined as ‘a carer’ or not), it was typical for the same professional to speak separately with the care‐recipient and carer, which was viewed to be the best approach to allow each person to speak confidentially and openly, and the information was routinely combined to form a broader contextual view of the dyad's needs.

So it's not just looking at the carer's assessment on its own, it's about taking that joint approach. I feel it helps when you're allocated to the person, because you already know the person's life, so, therefore, when you're having that discussion with the carer, you've already taken on board whatever knowledge you have already of the person. So, it sort of gets incorporated, embedded into one.

(PS23 social work apprentice)

That's what we do within our project. It is a family approach. So when we make contact with a carer, we then see what we can do to help with the cared for. Quite often, if you can get the right support in for the cared for, then the carer has a lot of that pressure alleviated.

(PS12 care provider)

Another perceived benefit of a dyadic approach to practice included the view that it helps to build trust when professionals are involving carers in the whole process of needs assessment, care planning and interventions, especially on an equal footing to the care‐recipient:

It's about your ability to spend time and get to know and understand an individual and their family. You develop that trust… They'll be honest with you, they'll open up to you; and where people do, it does make such a difference.

(PS2 carers’ organisation)

However, despite the overall positive views expressed about applying a dyadic outcomes approach, one respondent initially struggled to identify any benefits:

I can't immediately think of any, quite frankly. In some ways I feel it is better to separate them as individuals, because you can be very clear about your outcomes.

(PS18 LA staff)

#### 3.2.2 Challenges and barriers

- Recognising carers on an equal footing to care‐recipients

A commonly raised concern was that a dyadic approach may compound the existing tendency for the needs of carers' to be overlooked or considered as secondary to those of the care‐recipient (*n* = 15). Joint assessments (i.e. those conducted with both carer and care‐recipient, together) may not allow either party (but especially, carers) sufficient space to express their needs and views. Conducting separate assessments by the same professional, who then combines the information to form a broader view, was preferred as a way of balancing both individual and relational needs/outcomes. Similar concerns and the view that a degree of separation is beneficial were also expressed with regard to joint care planning and funding of support:

There is a very clear separation of funding—you know, the purpose of it and the outcomes. Whereas if you're putting it into one plan, I don't know whether either one of the parties, needs could be somehow overlooked. Um, maybe one would take priority over the other.

(PS18 LA staff)

ii Workforce resourcing, support and skills

A dyadic outcomes approach, especially in assessment and care planning, but also in the evaluation and planning of service delivery, requires the collection and combination of QoL need/outcome information from care‐recipients and carers. A key challenge identified was the additional time and resource to achieve this, including support for staff in its initial implementation (e.g. using individual or group supervision, training) and ongoing practice. This may affect the implementation of the approach to ensure consistency and quality of experience.

In terms of resources and time, in terms of focusing on that wider network—it'd probably be a bit of a struggle if I'm honest.

(PS20 social worker)

Concerns were also expressed with regard to the level of skill, knowledge and experience required by staff to navigate emotionally difficult conversations and also retain and apply detailed information: *“You've got to definitely be compassionate. Yeah*, *and a good memory”* (PS12 care provider) and “*There's something around the skill of the person… it's knowing the appropriate time to approach these conversations*” (PS14 carers’ organisation). While the same skills are needed regardless of whether individual or dyadic, the dyadic approach adds a further layer of complexity (e.g. in combining complex information, weighing different perspectives and complex problem solving).

iii Data protection and confidentiality

Data protection, confidentiality, disclosure and consent were important considerations, especially where organisations were working primarily, or only, with carers:

When we're talking to carers, you have to make sure that GDPR [General Data Protection Regulation] has been adhered to—if they're talking about somebody else in a lot more depth, you have to find out about, does this person give you permission to talk about the whole situation.

(PS16 carers’ organisation)

Where the same practitioner is conducting assessments with both carer and care‐recipient, or where organisational databases (typically, LA databases) allow for linking of data, this is not a significant issue. However, the consideration of different perspectives is more difficult where two different organisations (typically, a LA and a carers' organisation) conduct separate needs assessments, since they tend not to have a shared dastabase and/or regular ongoing communication.

iv Barriers related to systems‐level factors

The limitations of adult social care funding, competing demands on resources and priorities and lack of available services, all contribute to making it difficult to achieve good QoL outcomes, which applies generally, whether individually or dyadically.

Funding… it costs more money to be able to do it. To enable an older person that's cared for or carer, to have a good quality of life, a fuller life, you need to put that support in, to at least re‐engage them back with their community.

(PS9 carers’ organisation)

The current system of funding and commissioning services also tends to reinforce ‘silos’, that is funding is allocated for services either for adults with support needs *or* carers. Organisations, such as carers support and other local voluntary organisations, are often obliged to compete against one another for a LA contract. This not only acts against collaborative partnership working between agencies, but also undermines dyadic approaches and, potentially at least, improved outcomes.

… commissioning drives, doesn't it, how services operate for sure. So that is I think could be the number one challenge that services are just not set up to work like that …

(PS14 carers’ organisation)

Staff from carers' organisations highlighted the concern of ‘mission creep’. Adopting a broader, more dyadic, view of outcomes may be in conflict with their organisational aims and purpose, that is to offer specialist support to carers. In turn, and in time, this may affect their ability to attract funding for services, especially from LA commissioners.

We were set up as carers' organisations. We are funded to be carer support organisations. Do funders want to fund another generic service?

(PS3 carers’ organisation)

In this context, commissioners, who have a duty under the Care Act to shape local markets, play an important role. The current commissioning model, as noted above, tends to separate services ‘for’ adults with support needs from services ‘for’ carers. Along with other strategic‐level leaders, commissioners may influence a shift towards a dyadic outcomes focus. To effect such a change in a complex and fragmented system, however, requires leadership and investment at all levels.

… it needs buy‐in at all stages, and I think within [place] we're still on that journey, but I think there is sufficient buy‐in at all levels to get to that position. It does really need that strong leadership, both from the top, also from the bottom.

(PS24 commissioner)

## 4 DISCUSSION

There is increasing recognition of the needs of older carers, as well as the importance of supporting care dyads (i.e. people with care needs and their carers, individually and together) to improve QoL outcomes (Henwood et al., 2018; Larkin et al., 2019; O'Rourke et al., 2021), even if there are gaps in the existing evidence base (Zhang et al., 2022). In this study, we sought to explore social care professionals' views of the needs of older carers and the potential benefits, challenges and barriers to applying a ‘dyadic QoL outcomes’ approach with older caring dyads. The study identified that there is relatively little focus on older carers in practice. The sector tends to conceptually separate young or young adult carers, up to 25 years, but then considers all other adult carers together, regardless of age group, despite the evidence of older carers' specific needs and characteristics (Greenwood & Smith, 2016; Larkin et al., 2019, 2022). Our findings indicate that older carers are not acknowledged as a distinct group in the data collected and used within the sector to inform, influence and guide policy and service development or care practice.

Our findings also indicate that there is an appreciation of potential benefits of, and support for, applying a dyadic outcomes approach in practice. Benefits included a better understanding the intertwined nature of individual social care‐related needs and outcomes (as explored in, for example, [Rand et al., 2017]), which may guide needs assessment, care‐planning, service planning, oversight, delivery and commissioning to focus on improving the lives of older carers and their relatives. Developing a broader view of both care‐recipients' and carers' needs and outcomes was also perceived to be beneficial in building trust and open communication between professionals and families. Perceived barriers and challenges were also identified. These included the resources and staff skill level to collect, interpret and apply dyadic outcomes information in practice, especially needs assessment, care planning and service delivery. Issues were also raised with regard to confidentiality, data protection and ensuring carers' needs are not eclipsed by the needs of the care‐recipient. Finally, there were challenges related to underinvestment and the fragmented nature of the adult social care system (Marczak et al., 2021) and commissioning models that encourage inter‐agency competition, rather than collaboration focussed on supporting people's QoL.

These issues are interrelated and form part of the current landscape and context of adult social care in England. Under the Care Act, LAs are responsible for assessing and addressing any eligible needs for both adults with support needs and their carers. However, organisations other than the LA (typically, carers' organisations) may be contracted to conduct assessments on their behalf. They will often only assess carers' needs and offer support with a focus on carers, apart from the care‐recipient, which enables them to be viewed as co‐clients on an equal footing to adults with support needs (Rand & Malley, 2014). Arguably, this separation reflects the Care Act's position of defining eligible needs for carers and care‐recipients, separately, and its aim to achieve parity for carers. However, it may also undermine an approach that (re)conceptualises ‘assessment’ and ‘support’ through the lens of the dyad, despite the ‘whole family’ approach set out in the guidance (Department of Health and Social Care et al., 2014).

Closer joint working between LAs and other agencies, systems for effective data sharing and regular communication between organisations may partially address the issues. However, even where the same organisation (whether LA or another organisation) conducts needs assessments, there is the need to ensure confidentiality and to navigate the complexity of adopting a ‘dyadic’ view. Often, the approach taken by practitioners (as described in their current practice) is to complete separate assessments for older carers and care‐recipients and then recombine the information using their judgement. This allows a balance between the individual's needs, whilst also recognising and accounting for the dyadic perspective. This process may be difficult to operationalise in formalised systems, especially where there is disagreement, tension or conflict between parties, as a key aspect of the process is the development of trust between professionals and families. It would also require additional time, skill and resource, especially in navigating conversations with both parties in the caring dyad to develop a holistic view of needs and applying this in effective care planning.

To implement a dyadic approach in practice, there would need to be a re‐framing of needs assessments, care planning, commissioning and service delivery, so that QoL outcomes are not only considered individually, but also dyadically. Here, a key barrier is the unresolved issue of the conceptual and actual position of carers as both ‘co‐workers’ and ‘co‐clients’, as well as ‘free resources’ (Marczak et al., 2021; Rand & Malley, 2014). These competing tensions are impossible to reconcile in practice (Scourfield, 2005). In this study, it was suggested that this challenge may be partially alleviated by effective mid‐level and strategic‐level leadership that promotes a dyadic focus, especially by commissioners, who play an important role in setting the overall strategic direction, expectations of partnership‐working and allocating resources to care providers. However, especially in light of issues related to increased demand for long term social care, under‐funding and limited lack of effective integration of the long‐term care sector, which have been exacerbated by the pandemic (Department of Health and Social Care, 2021), systems‐level tensions persist that continue to undermine the adoption of a dyadic approach.

The study has some limitations. As a qualitative study focussed on LAs, care providers and carers' organisations in London, South East and central England, the findings offer insight into the views of professionals working in the social care sector. The sample included professionals working within diverse areas, in terms of urban/rural and levels of deprivation, and in a range of roles. Therefore, even though the study offers exploratory insights into professionals' views on supporting older carers and the dyadic outcomes approach, the regional focus of the study may overlook potential differences with other regions in England, across the UK countries or internationally. However, it is likely that the key issues identified in this study will have international resonance, especially in relation to the invisibility of older care dyads in research, policy and practice (Zhang et al., 2022) and late development of carer policy and support (Brimblecombe et al., 2018). Therefore, the study is likely to offer insights to inform and guide further research on dyadic approaches, in the UK and internationally.

## 5 CONCLUSION

This study offers insights into the perceived feasibility, benefits and challenges of applying a dyadic outcomes approach in practice. There was an overall appreciation of the potential benefits, especially in developing a holistic view of QoL‐related needs/outcomes for both care‐recipient and carer and also building trust between professionals and families. Barriers to applying a dyadic approach included data protection and sharing, both within and between organisations; the required workforce skills, experience and knowledge; and insufficient and competition‐oriented adult social care funding that discourages collaboration between agencies. Despite the potential of the approach to improve the effectiveness of support for older caring dyads, these challenges need to be recognised and addressed if a dyadic outcomes approach is to be successfully implemented in practice.

### AUTHOR CONTRIBUTION

SR and AM designed the study. SR conducted data collection and drafted the manuscript. SR, WZ, GC and BS conducted the data analysis. All authors contributed to the interpretation of results, provided critical feedback on the draft manuscript and approved the final manuscript.

## FUNDING INFORMATION

This report is independent research funded by the National Institute for Health Research School for Social Care Research (NIHR SSCR) (Grant Reference Number: 23063). The views expressed in this publication are those of the authors and not necessarily those of the NIHR SSCR, the National Institute for Health Research or the Department of Health and Social Care.

## CONFLICT OF INTEREST

The authors have no conflicts of interest to declare.

## Data Availability

Research data are not shared.
